# Supplementary material for: Cognitive Engagement and Subjective Well-Being in Adults: Exploring the Role of Domain-Specific Need for Cognition
Source: J Intell. 2024 Nov 3;12(11):110. doi: 10.3390/jintelligence12110110 (PMC11595623; doi:10.3390/jintelligence12110110)
Supplement: Supplementary file 1 [file jintelligence-12-00110-s001.zip › jintelligence-3194332-supplementary.pdf]

Supplementary Material. Intercorrelations of Well-Being Indicators.

Article: Cognitive Engagement and Subjective Well-Being in Adults: Exploring the Role of Domain-Specific Need for Cognition

Table S1. Intercorrelations Sample 1

							Study-Related Satisfaction					
	NFC$_{general}$	NFC$_{stud}$	NFC$_{job}$	PA	NA	LS	Subject	Conditions	Coping with Stress	JS	Self-Control	Reappraisal
NFC$_{study}$	.71***											
NFC$_{job}$	.71***	-										
Positive Affect	.29***	.25***	.39***									
Negative Affect	-.06	-.08	-.01	-.11*								
Life Satisfaction	.09	.16**	.21**	.37***	-.34***							
Study Satisfaction												
Subject-Related	.31***	.36***	-	.41***	-.21***	.38***						
With Conditions	-.03	.01	-	.11	-.17**	.23***	.33***					
Coping with Stress	.07	.12		.20**	-.31***	.37***	.28***	.41***				
Job Satisfaction	.11	-	.25**	.28***	-.13	.38***	-	-	-			
Self-Control	.25***	.13*	.27***	.40***	-.25***	.33***	.21***	.04	.09	.27***		
Reappraisal	.21***	.16**	.22**	.31***	-.15***	.26***	.25***	.06	.08	.07	.13**	
Suppression	.11*	.07	.01	-.12**	.13**	-.12*	-.10	-.07	-.05	-.11	-.04	.03

Note. Spearman rank correlations. N = 451. Student subsample n = 258. Working subsample n = 159. NFC$_{general}$ = domain-general Need for Cognition. NFC$_{domain}$ = study-specific Need for Cognition or job-specific Need for Cognition. PA = positive affect. NA = negative affect. LS = Life satisfaction. JS = job satisfaction

* p < .05. ** p < .01. *** p < .001.

Table S2. Intercorrelations Sample 2

							Study-Related Satisfaction					
	$NFC_{general}$	NFC_{study}	NFC_{job}	PA	NA	LS	Subject	Conditions	Coping with Stress	JS	Self-Control	Reappraisal
NFC_{study}	.66***											
NFC_{job}	.76***											
Positive Affect	.31***	.25**	.28***									
Negative Affect	-.02	-.10	.06	-.23***								
Life Satisfaction	.16*	.15	.22**	.46***	-.35***							
Study Satisfaction												
Subject-Related	.16*	.25**	-	.36***	-.21**	.48***						
With Conditions	.01	0.02	-	.04	-.05	.24**	.13					
Coping with Stress	.16*	.22**	-	.28***	-.26***	.39***	.20*	.42***				
Job Satisfaction	.26**	-	.25**	.31***	-.21**	.43***	-	-	-			
Self-Control	.11*	.20*	.03	.37***	-.31***	.35***	.30***	.04	.15	.17*		
Reappraisal	.16**	.09	.14	.28***	-.16**	.22***	.12	.09	.16*	.09	.22***	
Suppression	.04	.01	-.02	-.24***	.14**	-.26***	-.09	-.01	.02	-.16*	-.13*	-.12*

Note. Spearman rank correlations. N = 355. Student subsample n = 157. Working subsample n = 156. $NFC_{general}$ = domain-general Need for Cognition. NFC_{domain} = study-specific Need for Cognition or job-specific Need for Cognition. PA = positive affect. NA = negative affect. LS = Life satisfaction. JS = job satisfaction.

* $p < .05$. ** $p < .01$. *** $p < .001$.

Table S3. Intercorrelations Sample 3

	$NFC_{general}$	NFC_{job}	Positive Affect	Negative Affect	Life Satisfaction	Job Satisfaction	Self-Control	Reappraisal
NFC_{job}	.82***							
Positive Affect	.36***	.38***						
Negative Affect	-.06	-.09	-.18***					
Life Satisfaction	.17**	.24***	.51***	-.38***				
Job Satisfaction	.20***	.33***	.36***	-.31***	.46***			
Self-Control	.20***	.26***	.46***	-.31***	.35***	.24***		
Reappraisal	.12*	.13*	.30***	-.12*	.24***	.04	.22***	
Suppression	.02	-.02	-.16*	.06	-.17**	-.14*	-.06	.13*

Note. Spearman rank correlations. N = 346. $NFC_{general}$ = domain-general Need for Cognition. NFC_{job}= job-specific Need for Cognition.

* $p < .05$. ** $p < .01$. *** $p < .001$.
